# Educational supervision to support pharmacy professionals’ learning and practice of advanced roles

**DOI:** 10.1007/s11096-022-01421-8

**Published:** 2022-05-16

**Authors:** Michelle Styles, Helen Middleton, Ellen Schafheutle, Matthew Shaw

**Affiliations:** 1 Centre for Pharmacy Postgraduate Education (CPPE), Division of Pharmacy and Optometry, School of Health Sciences, The University of Manchester, Manchester, England; 2 Centre for Pharmacy Workforce Studies, Division of Pharmacy and Optometry, School of Health Sciences, The University of Manchester, Manchester, England

**Keywords:** Advanced practice, Clinical supervision, Pharmacy education, Primary care, Workplace-based learning

## Abstract

Pharmacy professionals are increasingly moving into advanced roles, including in primary care. In England, the publicly funded Pharmacy Integration Fund (PhIF) enabled employment and training of pharmacy professionals in new patient-facing roles, including general practice and care homes. In recognition of the need for support and supervision during work-based learning and building on established support structures in medicine and nursing, one of the providers of PhIF funded learning developed a supervision structure which mirrors arrangements for postgraduate medical specialty training. This paper describes what informed this supervision model, with a particular focus on educational supervision, its delivery, and the training which was developed to support supervisors. This supervision enabled pharmacy professionals moving into primary care to practise safely, manage workplace challenges, extend their roles and make progress with their education. This model illustrates the benefits of supervision in supporting post-registration learning to facilitate the development of advanced patient-facing clinical roles.

## Introduction

UK pharmacists train for 4 years at Masters level, followed by a supervised foundation training year in practice. Until recently, there has been little structured equitable access to post-foundation training, with pharmacists working in NHS settings most likely to enrol on funded training (such as postgraduate diplomas). Simultaneously, there has been a move to increase pharmacy professionals’ (pharmacists and pharmacy technicians) contributions to patient care to address increasing patient demand alongside workforce shortages. Benefits of advanced clinical roles for pharmacy professionals are: improving patients' access to healthcare [1, 2], reducing healthcare and prescribing costs [3, 4] and clinical interventions [5, 6]. In 2015, NHS England (NHSE) announced a pilot to support the development of clinical pharmacists in general practice (family medicine) to work “in a patient-facing role to clinically assess and treat patients using their expert knowledge of medicines for specific disease areas” [7, 8]. Four-hundred-and-sixty pharmacists enrolled on this pilot in 2015–2016 [9].

In 2016, NHSE launched the Pharmacy Integration Fund (PhIF) to accelerate integration of pharmacy professionals across primary care and committed to train an additional 1500 pharmacists in general practice. In 2018, NHSE expanded pharmacy roles into care homes, and a further 600 pharmacy professionals were recruited.

Under PhIF, pharmacy professionals were employed in primary care, where they fulfilled new patient-facing roles. With the link between high quality education and improved patient safety highlighted in the Francis Report [10], and to ensure consistency in standards of patient care, pharmacy professionals employed in NHSE-funded roles were required to enrol on an 18-month workplace-based education pathway. Health Education England (HEE) commissioned the Centre for Pharmacy Postgraduate Education (CPPE) to develop and deliver training for the 2015 pilot and subsequent pathways.

A longitudinal evaluation of the pilot found that pharmacists perceived improved competence [9] and this informed design of the initially separate pathways for general practice and care homes, later combined into one primary care pharmacy education pathway. These 18-month education pathways involved a range of learning modules, and those not already qualified could add a PhIF-funded independent prescriber course. Similar schemes were introduced in other UK home nations including Scotland [11] and Wales [12]. Recognising that pharmacy professionals were moving into new, patient-facing roles in new settings, CPPE designed a model of supervision to ensure progression and application of learning in practice.

This paper aims to describe what informed the development of the supervision model accompanying these pathways in England, and to describe the training developed to support supervisors. These descriptions are intended to inform ongoing discussions and planning for supported post-registration pharmacy practice.

## Support for workplace-based learning

Internationally, most healthcare professions such as medicine and nursing have formal support structures to facilitate workplace-based learning and safe practice [13–16] including mentors, preceptors and clinical supervisors [17]. Supervision has been defined as “the provision of guidance and feedback on matters of personal, professional and educational development in the context of a trainee’s experience of providing safe and appropriate patient care” [18]. Evidence suggests that supervision has a positive effect on patient outcomes and trainee development, and provides opportunities for focussed, constructive feedback [19, 20]. While preceptor supervision of trainee and novice pharmacists during periods of learning in practice is common worldwide, few countries have formal structures to support more advanced workplace-based learning in pharmacy and there is a need for a structured and evidence-based approach to preceptor training and supervision in pharmacy [21, 22].

CPPE’s workplace-based education pathways mirror arrangements for postgraduate medical specialty training which uses an experiential learning model where trainees develop skills and knowledge while delivering patient care in the workplace [23]. Workplace-based learning has traditionally been part of pre-registration (now foundation) pharmacy training, supported by work-based tutors [24]. Evidence exists that pre-registration trainee experience and outcomes vary in different settings and sectors [25–27]. In the UK, pharmacist post-registration learning is usually self-directed without formal support structures, except when training as an independent prescriber, where workplace-based supervision by a designated prescribing practitioner is mandated.

**Fig. 1:**
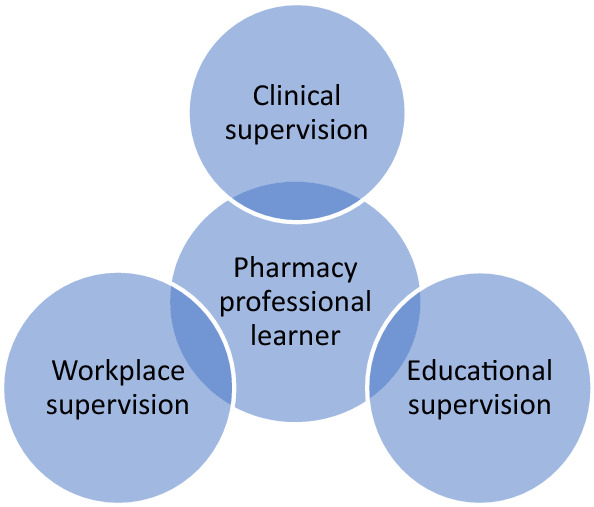
CPPE learner supervision support structures for pharmacy professionals

Recognising the lack of infrastructure for pharmacy professionals to support workplace-based learning for advanced roles [28, 29], and to support development of application in practice and self-directed learning [30], CPPE established a learner supervision structure to underpin the pathway and support learners to acquire the knowledge, skills and competencies to practise safely. This support structure includes clinical and educational supervision as well as workplace supervision to mirror postgraduate medical specialty training [18, 31, 32] (Fig. 1). This structure recognises that learning comes from a combination of formal learning activities and self-directed private study, with the most important element being the opportunity to apply learning in practice, under appropriate supervision.

## Clinical supervision

Clinical supervision forms an integral part of post-registration training in medicine and nursing, particularly within the context of new and emerging roles. Clinical supervisors usually work alongside trainees in day-to-day practice and are responsible for safeguarding the quality of patient care and undertaking workplace-based assessments [33]. The aim of clinical supervision is to build confidence, capability, clinical reasoning, and critical thinking [34, 35].

As many of those recruited to primary care roles have little experience of the sector and lack the necessary clinical assessment skills [36, 37], clinical supervision was provided to assure quality of care, to help pharmacy professionals integrate into the primary care environment, and to facilitate role development. In the case of pharmacists, the clinical supervisor was a general practitioner (GP—family doctor), whereas for pharmacy technicians, the clinical supervisor was a senior pharmacist.

Recognising the importance of pharmacists experiencing other pharmacists in practice, senior pharmacists were also recruited into primary care settings to provide workplace supervision [38].

## Educational supervision

Educational supervision in postgraduate healthcare training appears to be unique to the UK [39]. It is concerned with supporting and monitoring trainee progress throughout a training programme, to ensure that trainees are exposed to appropriate clinical experience and responsibility, with support and oversight [18, 40]. Educational supervision is less prevalent than clinical supervision, with formal structures in place only for medicine, and more recently, for pharmacy and advanced clinical practice [34]. Educational supervision in pharmacy has been shown to impact positively on the ability of learners to manage workplace challenges and develop their roles (Styles, unpublished report), and can also help trainees to develop self-confidence [41].

Educational supervision was considered an important part of the CPPE supervision structure, as most of those recruited to primary care roles were unfamiliar with both the general practice environment [9] and the processes required for workplace-based learning [30]. CPPE education supervisors were appointed to oversee the developmental trajectory of pharmacy professionals enrolled on the pathways and ensure they have an effective learning experience. Like postgraduate medical educational supervisors, CPPE education supervisors work with learners to help them identify learning and development needs and support them to produce personal development plans (PDPs). CPPE education supervisors also conduct progress reviews, track assessments and provide feedback in relation to PDP goals and role progression.

## Development of training for CPPE education supervisors

Recognising the lack of experience of many pharmacy professionals to act as effective supervisors, CPPE developed a training programme for education supervisors. Content was based on the Academy of Medical Educators (AOME) [42] framework for the professional development of postgraduate supervisors, with most competencies for medical educational supervisors considered equally relevant to CPPE education supervisors. Table 1 lists the identified learning needs of CPPE education supervisors, omitting framework areas 1 and 2 of the AOME framework [42] which focus on clinical competencies, as CPPE education supervisors are not required to be clinicians.

**Table 1:** Learning needs identified for CPPE education supervisors

Academy of Medical Educators [42] framework area	Learning needs identified for CPPE education supervisors
Framework Area 3: Teaching and facilitating learning	∙ Supporting learners to undertake self-directed learning ∙ Supporting the learner to progress in their role ∙ Facilitation of workshops and small group learning
Framework Area 4: Enhancing learning through assessment	∙ Planning and monitoring assessment activities ∙ Giving honest and constructive feedback ∙ Marking reflective essays ∙ Portfolio review ∙ Professional discussions regarding 360-degree feedback ∙ Supporting learners to prepare for assessments
Framework Area 5: Supporting and monitoring educational progress	∙ Supporting learners with learning needs analysis and personal development planning ∙ Reviewing and monitoring learner progress ∙ Awareness of, and able to access available support for practitioners requiring additional support (PRAS)
Framework Area 6: Guiding personal and professional development	∙ Role modelling and mentorship ∙ Supervisory conversational skills

The first induction training programme for CPPE education supervisors comprised two workshops:

*Mentoring, feedback and workplace-based assessment* focused on supporting self-directed learning, interpersonal skills for supervisory conversations, coaching to structure mentoring conversations, giving constructive feedback and the principles of workplace-based assessment.

*Pathway supervision* focused on learning needs analysis, reviewing progress with learning, role plays to develop supervisory conversational skills and supporting practitioners requiring additional support [43].

In addition, education supervisors participated in two webinars where they were trained to mark reflective essays and participated in assessor standardisation activities. Education supervisors also participated in a 3-day residential course to develop facilitation and presentation skills which is an established part of the induction process for all CPPE staff.

## Changes to the CPPE education supervisor training programme

Induction training for education supervisors has evolved since the initial iteration as additional learning needs were identified. Leadership training was introduced, aligned to the Holding to Account and Influencing for Results domains of the NHS Healthcare Leadership Model [44]. This was to support education supervisors when learners were not engaging in the education pathway or where there was poor conduct at workshops.

Informed by research that showed learners valued peer interactions (Styles, unpublished report) as well as one-to-one support [45], individual supervision was replaced with group tutorials and supervisors were provided with training on group dynamics and facilitating group tutorials.

Future changes to supervisor training include additional training on portfolio assessment due to the introduction of a core advanced pharmacist curriculum [46].

## Adaptations for the COVID-19 pandemic

In 2020, the supervision model was adapted in response to the COVID-19 pandemic. Learner workshops were repurposed for online delivery, and education supervisors were supported to develop competence in facilitating in the online environment by the introduction of online facilitation skills training underpinned by an e-course. Online workshops were well received by participants and education supervisors perceived that they afforded opportunities for learners to expand professional networks beyond their locality [47]. Workshops will remain online except where there is benefit in learning practical skills.

## Quality assurance of education supervisors

In 2019 an annual review of practice for CPPE education supervisors was introduced for quality assurance. Line managers observe supervision sessions and assess education supervisors in relation to good practice criteria derived from the AOME framework for the professional development of postgraduate supervisors [42].

## Discussion

Pharmacy professionals are increasingly moving into patient-centred, advanced practice roles, with significant investments made in England via the NHS Pharmacy Integration Fund (PhIF). Evidence is emerging that PhIF learning pathways achieved their intended outcomes of pharmacy professionals working in advanced roles in primary care, with increased confidence and autonomy positively impacting patient care [9, 48, 49]. It is critical that NHSE continues to fund training to facilitate pharmacy professional integration into these new roles [48].

Supervision is an important part of learning for pharmacy professionals, as it enables application of learning in practice, facilitates competence in advanced skills, and supports confidence to use these skills in direct patient care [29]. Whilst supervision is an established part of post-registration learning and practice for other healthcare professions including medicine and nursing, comparable processes and infrastructure are lacking in pharmacy.

This paper has summarised CPPE’s experience of establishing a model for supervision on some PhIF-funded primary care pathways, and training that has been put in place to support supervisors. Clinical supervisors and designated prescribing practitioners (DPPs) were commonly GPs, but as more pharmacists complete the pathway and gain experience as DPPs, the competencies attained may make them suitable as clinical supervisors in future. In the interim, work-based supervisors and/or clinical mentors can help to address the lack of understanding of pharmacy professionals’ contribution in primary care and ensure exposure to pharmacy role models.

This paper has focussed on the role of education supervisors, who oversee the period and process of learning, and the training offered to them. Indeed, the role of education supervisor may be particularly relevant in settings where clinical supervision is more difficult to facilitate, such as urgent care settings and community pharmacy [49, 50].

The model of (educational) supervision developed by CPPE and described in this paper can likely inform plans for continuous and lifelong learning which needs to become more embedded within pharmacy at all career stages, and in all settings. These insights will be relevant for current changes under the new General Pharmaceutical Council education and training standards, where work-based learning will be important to ensure pharmacists are sufficiently clinically trained and prescriber ready after 5 years (from 2026).
